# A Microarray Study of Carpet-Shell Clam (*Ruditapes decussatus*) Shows Common and Organ-Specific Growth-Related Gene Expression Differences in Gills and Digestive Gland

**DOI:** 10.3389/fphys.2017.00943

**Published:** 2017-11-28

**Authors:** Carlos Saavedra, Massimo Milan, Ricardo B. Leite, David Cordero, Tomaso Patarnello, M. Leonor Cancela, Luca Bargelloni

**Affiliations:** ^1^Instituto de Acuicultura Torre de la Sal, Consejo Superior de Investigaciones Científicas, Castelló de la Plana, Spain; ^2^Dipartimento di Biomedicina Comparata e Alimentazione, Universitá di Padova, Polo di Agripolis, Legnaro, Italy; ^3^Centre of Marine Sciences (CCMAR), Universidade do Algarve, Faro, Portugal; ^4^Department of Biomedical Sciences and Medicine and Academic Biomedical Centre, Universidade do Algarve, Faro, Portugal

**Keywords:** growth rate, organ-specific gene expression, digestive gland, gills, bivalves, insulin signaling pathway

## Abstract

Growth rate is one of the most important traits from the point of view of individual fitness and commercial production in mollusks, but its molecular and physiological basis is poorly known. We have studied differential gene expression related to differences in growth rate in adult individuals of the commercial marine clam *Ruditapes decussatus*. Gene expression in the gills and the digestive gland was analyzed in 5 fast-growing and five slow-growing animals by means of an oligonucleotide microarray containing 14,003 probes. A total of 356 differentially expressed genes (DEG) were found. We tested the hypothesis that differential expression might be concentrated at the growth control gene core (GCGC), i.e., the set of genes that underlie the molecular mechanisms of genetic control of tissue and organ growth and body size, as demonstrated in model organisms. The GCGC includes the genes coding for enzymes of the insulin/insulin-like growth factor signaling pathway (IIS), enzymes of four additional signaling pathways (Raf/Ras/Mapk, Jnk, TOR, and Hippo), and transcription factors acting at the end of those pathways. Only two out of 97 GCGC genes present in the microarray showed differential expression, indicating a very little contribution of GCGC genes to growth-related differential gene expression. Forty eight DEGs were shared by both organs, with gene ontology (GO) annotations corresponding to transcription regulation, RNA splicing, sugar metabolism, protein catabolism, immunity, defense against pathogens, and fatty acid biosynthesis. GO term enrichment tests indicated that genes related to growth regulation, development and morphogenesis, extracellular matrix proteins, and proteolysis were overrepresented in the gills. In the digestive gland overrepresented GO terms referred to gene expression control through chromatin rearrangement, RAS-related small GTPases, glucolysis, and energy metabolism. These analyses suggest a relevant role of, among others, some genes related to the IIS, such as the ParaHox gene *Xlox*, CCAR and the CCN family of secreted proteins, in the regulation of growth in bivalves.

## Introduction

Growth is one of the most characteristic features of biological entities, and has a significant importance for food production. The characterization of the molecular, cellular, and organismic events that underlie growth has progressed considerably, especially in mammals and model organisms such as Drosophila (Andersen et al., 2013; Gokhale and Shingleton, 2015). However, non-model organisms, especially invertebrates, are far from this level of understanding. While it is clear that many molecular processes (e.g., signaling pathways) that act at the level of cell growth, cell division, and organogenesis are common to mammals and flies, and therefore should be also acting in other zoological groups as well, the fact that mammals and insects show usually determinate growth (i.e., growth stops when they reach a certain size) while most other invertebrates usually do not (Sebens, 1987; Charnov et al., 2001) suggests that some fundamental aspects of growth regulation will be different.

Bivalve mollusks make an interesting experimental material for studying growth in invertebrates with indeterminate growth. Growth can be measured easily and precisely in bivalves thanks to the presence of a hard shell, and usually their growth can be described by von Bertalanffy models (Urban, 2002; Dexter and Kowalewski, 2013). On the other hand, some bivalve species are farmed, which facilitates access to culture facilities and genomic resources. Since several mollusk species are important as food, the study of the growth rate differences among individuals and species has a special relevance in this zoological group because it can help in designing adequate husbandry procedures and to increase yield (Wilbur and Owen, 1964).

The study of growth variability in bivalves has been approached from several disciplines (Gosling, 2015). It has been clearly established that environmental variables, such as the temperature, and the availability of food influence the individual growth rate of marine mollusks (Figueiras et al., 2002; Tamayo et al., 2011). From a genetic perspective, individual growth rate in bivalves is often positively correlated with the degree of heterozygosity exhibited by enzyme coding genes (Zouros, 1987; Szulkin et al., 2010). This multilocus heterozygosity-growth correlation explains usually 2–10% of the variation in growth rate across individuals. The heterozygosity-growth correlation has been studied from a physiological perspective, and results have shown that more heterozygous, faster growing individuals are characterized by higher protein turnover rates and higher metabolic rates (Bayne and Hawkins, 1997). The abundance of somatic aneuploidy has been also related to differences in growth rates in bivalves (de Sousa et al., 2011). Quantitative genetics studies have shown that growth rate has a significant genetic component, with heritabilities rising often over 0.4 (e.g., Wang et al., 2010; Kong et al., 2015; Guiñez et al., 2017). Selective breeding for increased growth rate has been usually successful (e.g., Rodrigues De Melo et al., 2016). Genomic approaches have been used to detect the quantitative trait loci (QTL) underlying variability in growth rate (Jiao et al., 2013; Nie et al., 2017).

The molecular details of these observations, and in general of the growth rate differences among species and individuals, have not been adequately explored. The detection in mollusks of the basic elements of endocrine and regulatory networks of vertebrates point to a similarly complex system underlying growth differences in this group of organisms. Glimpses of this complexity have been collected through a number of studies. Three neuropeptides secreted in neural ganglia in abalones have been shown to be correlated with growth rates (York et al., 2012), supporting the existence of a neural control of growth. The role of insulin-related peptides in control of growth in mollusks has also been shown by studying the association of genetic polymorphisms with differences in growth (Kellner-Cousin et al., 1994; Gricourt et al., 2003; Shipilov et al., 2005; Wang et al., 2008; Cong et al., 2013; Feng et al., 2014; Alarcon-Matus et al., 2015). Molecules involved in growth regulation in vertebrates, such as myostatin, show similar roles in bivalves (Núñez-Acuña and Gallardo-Escárate, 2014; Morelos et al., 2015). The effect on growth rate of the polymorphisms in the genes coding for some proteins involved in nutrient acquisition, such as amylases, has been shown in oysters (Prudence et al., 2006).

The developments in genomics and proteomics allow now a more detailed and complete study of the molecular physiology of growth in bivalves and other mollusks. For example, a QTL study carried out in the scallop *Chlamys farreri* has implicated a gene coding for a growth factor receptor protein in determining growth rate variation in this species (Jiao et al., 2013). Transcriptomics, the branch of genomics that deals with genome-wide patterns of gene expression, can also contribute importantly to the understanding of the physiological and molecular basis of growth in these organisms. While many transcriptomic studies of mollusks have been published, only a few have focused on growth in bivalves. Hedgecock et al. (2007) and Meyer and Manahan (2010) studied oyster larvae obtained from reciprocal crosses between two inbred lines that showed heterosis for growth (i.e., they grew faster than both parental lines). They used massive parallel sequencing to determine the differences in gene expression between the parental lines and the hybrid lines, and they found a set of genes whose expression pattern was heterotic (i.e., they showed higher expression in the hybrid offspring than in the inbred offspring). Many of these genes were shown to be ribosomal proteins. In another study, Shi and He (2014) performed RNA-Seq on large and small farmed pearl oysters and confirmed differential expression associated to faster growth for 19 genes using qPCR. Among other mollusk taxa, only abalones, which are gastropods, have been the subject of transcriptomic studies of growth (van der Merwe et al., 2011; Choi et al., 2015; Valenzuela-Miranda et al., 2015). All together these studies indicate that differential expression associated to differential growth appears at a great variety of genes with very different functions.

Progress in the understanding of the physiological causes of growth variability in mollusks using transcriptomics can be achieved in several ways. A common feature of previous transcriptomic studies of growth in this group of organisms is that gene expression has been characterized in whole-animal samples. An exception is the study of Valenzuela-Miranda et al. (2015) in the abalone, which was focused on the foot muscle because it is the part of the body that has commercial value. However, different organs and tissues exhibit different expression patterns at a proportion of the genes as a result of their different functions (e.g., Milan et al., 2011; Moreira et al., 2015). Therefore, the use of whole-animal samples in transcriptomic studies of growth limits importantly the data and conclusions that can be drawn from those studies. While in some of the studies reported above the expression of a small set of selected genes was further analyzed in specific organs by quantitative PCR (van der Merwe et al., 2011; Shi and He, 2014; Choi et al., 2015), they represent a tiny fraction of the whole set of differentially expressed genes (DEGs) discovered, and therefore they offer only limited information on the molecular basis of organ-specific functions related to differential growth. The analysis of transcriptomic profiles of separate organs is clearly necessary. The digestive gland and the gills appear as two immediate candidates for detailed studies on growth. In Bivalves, the gills are involved in respiration and food selection, two of the main functions that influence growth. They also represent a first interacting front with the surrounding environment and therefore with potential pathogens, parasites, and toxins. The digestive gland is responsible for food digestion and storage of energy reserves, and probably has other less well characterized functions (Röszer, 2014). The potential importance of the gills and the digestive gland for growth in mollusks can be illustrated by the study of Tamayo et al. (2011), which showed that Manila clams featuring a higher scope for growth (a measure of the energy available for growth) had on average bigger gills and digestive glands.

A second way to progress is to move from observational studies, in which no null hypothesis is tested and conclusions are drawn from a review of the results, to hypothesis-driven studies based on the accumulated scientific knowledge. While an observational approach is valid, current understanding of animal growth processes at the molecular and cellular levels allow for constructing specific null hypothesis that can be tested in transcriptomic studies. Specifically, the research carried out in Drosophila, mouse and humans has allowed to identify a set of genes which are involved in the regulation of the molecular and cellular processes that underlie tissue and organ growth and size control (reviewed in Weinkove and Leevers, 2000; Lecuit and Le Goff, 2007; Yang and Xu, 2011; Andersen et al., 2013; Gokhale and Shingleton, 2015; Nijhout, 2015). This gene set will be referred to as the “growth control gene core (GCGC)” along this paper, and is made of six groups of functionally related genes. One group comprises the genes coding for growth hormones, insulin and insulin-like growth factors, and its receptors and receptor-associated proteins (proteins of the insulin/IGF-signaling pathway or IIS). In mollusks this group is represented by insulin-related peptides (ILP), their receptors and the receptor-associated proteins. IIS acts downstream through two main signaling pathways, one involving the Pi3K/Akt transduction cascade, and the other involving the Ras/Raf/MAPK signaling pathway. Another group of proteins senses the nutritional level of the cells and act to regulate the expression of growth factors, mainly through the target of rapamycin (TOR) signaling pathway. Another signaling pathway, involving JNK proteins, is sensitive to oxidative stress and other types of cellular stress, which are registered by TNFR, GPCR, and RTK receptors. Finally, the Hippo signaling pathway mainly processes the information provenient from cell-cell interactions. At the end of these signaling pathways, several transcription factors such as FOXO, eIF4E, or TEAD, regulate the expression of growth factors which control cell growth, cell proliferation and apoptosis. These are cell cycle regulators such as the cyclin-dependent kinases Cdk1 and Cdk4, Cyclin D, Cyclin E, and Myc. The GCGC provides a basic molecular setting in which differential gene expression related to growth rate differences can be sought. In mollusks, a taxon that has been poorly characterized with respect to the molecular aspects of growth control, a null hypothesis that establishes a concentration of differential expression at the genes belonging in the GCGC appears as a valid null hypothesis to be tested in any initial transcriptomic study of growth rate variation.

In this paper we report the results of such a study in the carpet-shell clam (*Ruditapes decussatus)*. *R. decussatus* is one of the five clam species of the subfamily Tapetinae that have commercial value in the NE Atlantic and in the Mediterranean Sea (Fischer-Piette and Métivier, 1971). The carpet-shell clam often exhibits a lower growth rate than other commercial clam species, which increases substantially the economic risks of its production, and makes the investigation of the molecular physiology of growth especially interesting in this species from a comparative perspective. We have improved a previously developed microarray (Leite et al., 2013) and applied it to study gene expression separately in the gills and the digestive gland of fast and slow-growing clams. We have searched for organ-specific or shared molecular functions related to differential growth, and we have tested the contribution of the GCGC to the growth-associated differential gene expression.

## Materials and methods

### Experimental set up and growth measurement

Fifty adult clams of ca. 30 mm were captured in February in Ria Formosa, near Faro (Portugal). Animals were labeled and transferred to the nearby Ramalhete Marine Station, where they were placed in a 400 L PVC tank located outdoors and supplied with running sea water pumped directly from the sea. The animals therefore had access to the same food that they ingested in their natural habitat. The tank was covered with a nylon mesh that let the air flow normally but kept the animals out of the reach of potential predators such as crabs and seagulls. Clams were inspected daily to check for mortality or stress symptoms. Temperature and salinity in the seawater was recorded daily.

Individual growth was recorded by measuring the increase in two dimensions of the shell. Measures of length and height were taken with a caliper at the onset and at the end of the experiment (12 weeks). The two measures were added up to give a single value of shell size. The growth rate for the study period was estimated as (S_f_ – S_i_)/S_i_, where S_f_ and S_i_ stand for final and initial shell sizes, respectively.

### Tissue sampling and RNA extraction

Clams were opened by cutting the adductor muscles with a scalpel blade, while keeping the animals on ice. Then the gills and the digestive gland were excised with the help of sterile forceps and scissors, and placed in separate 2 ml plastic tubes containing 1.5 ml of RNAlater® solution in ice. Digestive gland was minced before soaking. The tissues were let soak at 4°C for 12 h and then stored at −80°C until further processed.

RNA was extracted from each organ sample in each individual using Trizol following the protocol suggested by the manufacturer. An aliquot of RNA from each sample was run in an agarose gel to check for RNA quality and integrity. RNA concentration was measured with NanoDrop. The RNA was further purified with a QIAquick RNA Mini spin column and stored in TE buffer (10 mM Tris, 0.1 mM EDTA).

### Transcript annotation and DNA microarray design

Gene transcription analyses were performed using a 8 × 15 K Agilent oligo-DNA microarray platform deposited in the GEO database (http://www.ncbi.nlm.nih.gov/geo/) under accession number GPL23511. Briefly, a total of 41,119 contigs of *R.decussatus* obtained in the previous study (see Leite et al., 2013 for details) were reannotated to design a new version of *R. decussatus* DNA microarray. The Basic Local Alignment Search Tool (BLAST) was used to perform annotation of *R. decussatus* contigs. Batch Blast similarity searches for the entire set of contigs were locally conducted against NCBI (National Centre for Biotechnology Information) amino acidic non redundant (nr) database using Blastx option. To improve the number of annotated contigs five different approaches were attempted: (i) blastx searches (cut off *e*-value of < 1.0 E-3) against protein database UniProtKB/SwissProt, (ii) blastx (cut off *e*-value of < 1.0 E-3) and blastn (cut off *e*-value of < 1.0 E-5) searches against proteins and high quality draft trascriptomes of *Danio rerio, Gasterosteus aculeatus, Oryzias latipes, Takifugu rubripes, Tetraodon nigroviridis, Homo sapiens, Bos taurus, Mus musculus, Xenopus tropicalis, Drosophila melanogaster, Aedes aegypti, Aedes mellifera, Schistosoma mansoni, Caenorhabditis elegans, Ciona intestinalis, Ciona savignyi, Culex quinquefasciatus* available on Ensembl Genome Browser, (iii) blastx (cut off *e*-value of < 1.0E-3) and blastn (cut off *e*-value of < 1.0 E-5) searches against proteins, transcripts and assembly scaffolds *of Lottia gigantea* v1.0 database and *Crassostrea gigas*. The Gene Ontology (GO) terms associations for BP, MF, and CC were performed using Blastx algorithm against the NCBI amino acid nr database implemented in Blast2GO software.

Probe design started with the selection of target sequences to be represented onto the *R. decussatus* microarray. All annotated entries (13,161 corresponding to the 32% of total contigs) were included. Probe design was carried out using the Agilent eArray interface. Microarrays were synthesized in situ using the Agilent ink-jet technology. Each array included default positive and negative controls. A total of 14,003 probes, representing 13,161 *R. decussatus* transcripts were successfully obtained. Probe sequences and other details on the microarray platform can be found in the GEO database (https://www.ncbi.nlm.nih.gov/geo/) under accession number GSE99243-GSE99244 Microarrays were synthesized in situ using the Agilent non-contact ink-jet technology with a 8 × 15 K format and including default positive and negative controls.

### Microarray hybridization

Each microarray was hybridized with the RNA from a single individual for both organs (not pooled). Therefore, since five individuals were sampled in each growth class (F or S), five biological replicates were available in each organ/growth combination. Due to organizational reasons, hybridizations for gills were separated 6 months in time from hybridizations of digestive gland. Labeling of RNA samples and hybridizations were performed according to the Agilent Gene Expression Analysis protocols described by Milan et al. (2015). Processed slides were scanned at a 5-μm resolution using an Agilent G2565BA DNA microarray scanner. The default settings were modified to scan the same slide twice at two different sensitivity levels (XDR Hi 100% and XDR Lo 10%). Two linked images were generated for each slide. The data were extracted, and the background was subtracted using the standard procedures contained in AGILENT FEATURE EXTRACTION (FE) software, version 9.5.1. The software returns a series of spot quality measures in order to evaluate the goodness and the reliability of spot intensity estimates.

### Statistical analysis

All control features (positive, negative, etc.), except for Spike-in (Spike-in Viral RNAs), were excluded from subsequent analyses. Spike-in control intensities were used to identify the best normalization procedure for each dataset. After normalization, spike intensities are expected to be uniform across the experiments of a given dataset. Normalization procedures were performed using R statistical software using an in-house script. Quantile normalization always outperformed cyclic Lowess and quantile-normalized data were used in all subsequent analyses. For each tissue, only probes that provided values over the background threshold in at least two individuals were used for analysis. Fluorescence values that were lower than the background were substituted by the minimum significant value obtained in the whole set of probes of each tissue, which was 1.9 for gills and 2.8 for digestive gland.

Data were first analyzed through principal components and clustering methods to detect outliers, using the TMeV program suite (Saeed et al., 2003). Different clustering algorithms and correlation statistics were used with very similar results, and only average linkage clustering based on Pearson correlation coefficients will be reported.

Fluorescence values from each individual and tissue were tested for differences between F and S clams by a rank products procedure (Breitling et al., 2004). This non-parametric method takes the individual values for all genes in each group and ranks them. Then a test-statistic (the rank product) is computed. Finally, its associated probability (P) and the rate of false positive values (PFP), an analog of the false discovery rate, is computed by permutation of the individual group values and recalculation of the rank products. The method combines robustness to noisy data and small sample sizes with relatively high sensitivity (Breitling et al., 2004; Hong et al., 2006; Kadota and Shimizu, 2011). Calculations were carried out on the RankProduct web platform (Laing and Smith, 2010), which delivered as output the P and PFP values for two alternative null hypotheses: higher expression in F than in S (F>S) and higher expression in S than in F (S>F). PFP values < 0.15 were chosen as a threshold to select genes showing significant differential expression for further analyses (Breitling et al., 2004). Fold change (FC) estimations, expressed as the ratio of the average normalized fluorescence values for the S and F groups, were also provided by the program. Because FC estimates are based in a small number of animals, they are affected by wide variances and can be strongly biased. Therefore, they will be reported only to illustrate the observed expression differences between F and S groups but not to make inferences on the whole group of clams or on physiological processes. We will base or conclusions on P and PFP values.

### Functional characterization of differentially expressed genes

Gene Ontology (GO) terms were retrieved from the non-redundant (nr) and SwissProt protein databases using Blast2GO (Conesa et al., 2005). The GO terms obtained for the DEGs were subjected to a redundancy reduction procedure using REVIGO (Supek et al., 2011). The SimRel semantic similarity measure was used, small (0.5) similarity was allowed, and GO term sizes were obtained from the Uniprot database.

### Growth control gene core

Genes that have been demonstrated to be involved in the control of growth rate and body size in model organisms such as *Drosophila*, mouse and human, were obtained from the literature. We will refer to this gene set as the “gene control gene core” along the paper. Specifically, we used recent reviews of the topic to identify those genes (Weinkove and Leevers, 2000; Lecuit and Le Goff, 2007; Yang and Xu, 2011; Andersen et al., 2013; Gokhale and Shingleton, 2015; Nijhout, 2015). These genes are those involved in the insulin/insulin-like growth-factor axis, and their downstream signaling pathways PI3K/Akt and Ras/Raf/MAPK, but also genes coding for proteins in other signaling pathways related to growth control such as TOR, Hippo and JNK. Intermediate genes in these pathways which were not specifically cited in the reviews were searched through the Kyoto Encyclopedia of Genes and Genomes (KEGG) available at http://www.genome.jp/kegg/. Enrichment for this gene set within the whole set of DEGs was tested through the Fisher exact test.

### Growth-related genes

In a subsequent step, all the genes showing differential expression were scrutinized for their relationship with physiological and molecular processes related to growth as follows. Searches over the whole GO term set retrieved in the annotation step were performed for the terms growth, cell proliferation, cell cycle, cell division, tissue or organ development and differentiation. Genes related to energy metabolism and protein biosynthesis were also addressed, as they usually appear as differentially expressed between fast and slow growing mollusks in the literature (Meyer and Manahan, 2010). Genes whose gene expression was related to growth variation in specific articles dealing with mollusks (see section Introduction) were also individually searched in the annotated set of DEGs. Only genes that could be identified by Blastx at Eval < 1.0E-03 will be reported.

### GO term enrichment tests

A more systematic functional interpretation of differentially transcribed genes was obtained through enrichment analysis using Database for Annotation, Visualization, and Integrated Discovery (DAVID) software (Dennis et al., 2003; Huang et al., 2009) and considering GO Biological Process Database and KEGG pathways. DAVID software allows functional annotation of DEGs through enrichment analyses based on an integrated biological knowledgebase, containing over 40 annotation categories. Since DAVID databases contain functional annotation data for a limited number of species, it was necessary to link *R. decussatus* transcripts with sequence identifiers that could be recognized in DAVID (e.g., Ensembl Human and Zebrafish Gene IDs). This was carried out using dedicated Blast searches performed with Blastx (*E*-value < 10-3). Two alternative strategies were tested: in the first case, *R. decussatus* entries were matched to human Ensembl Gene IDs, while in the second strategy *R. decussatus* entries were associated with zebrafish Ensembl Gene IDs. As reported by Milan et al. (2011) the second strategy allowed the assignment of a putative homolog to a larger number of clam transcripts. Zebrafish IDs corresponding to differentially expressed transcripts and to all genes represented on the array were obtained from the corresponding Ensembl protein entries using the BIOMART data mining tool (http://www.ensembl.org/biomart/martview) and were then used to define a “gene list” and a “background” in DAVID, respectively. DAVID settings were gene count = 2 and ease = 0.1.

## Results

### Survival and growth rates

At the end of the experiment, 40 clams conserved the identification mark, of which 17 had died and 23 were alive (58 % survival rate). Mortality took place in the last week of the study period. Individual growth rates in the 12-week period of study varied between 1.2 and 23.7% (average 8.0 ± 0.8%). The average initial sizes of the live and dead animals were 46.4 ± 2.9 and 47.3 ± 4.4 mm, respectively. Dead animals showed an average growth rate of 6.2 ± 1.0%, and those that survived showed a growth rate of 8.9 ± 1.1%. These values indicate that neither initial size nor growth rate were statistically different between the groups of dead and live clams.

**Figure 1:**
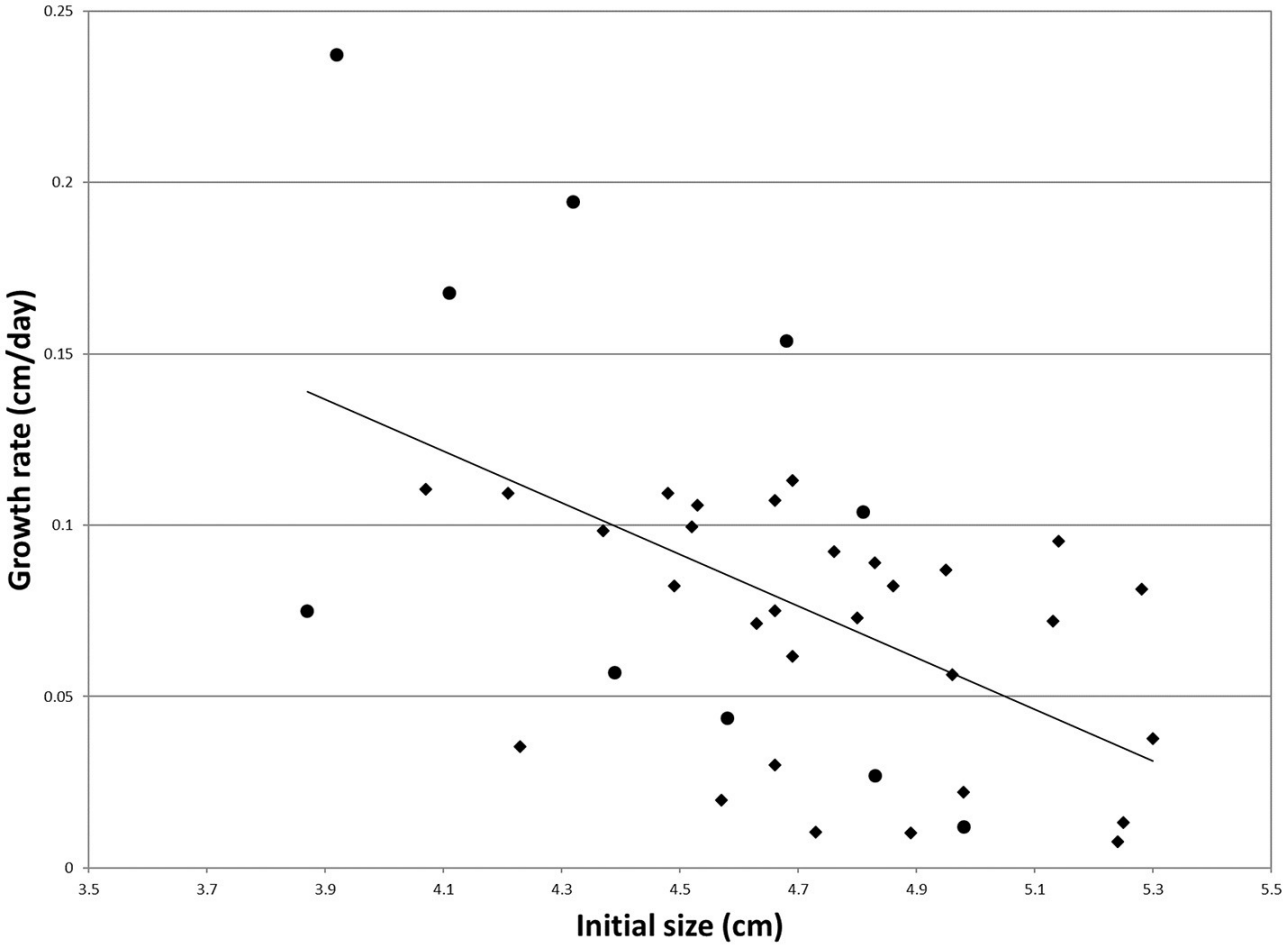
Individual growth rate regressed onto the initial size for the clams of the experimental population (*N* = 40). Dots mark the individuals that were used for gene expression analysis. The regression equation is as follows: *y* = −0.0753*x* + 0.4303 (*R*^2^ = 0.28).

Regression of individual growth rates on the initial size was significant (Figure 1). Individual residuals were therefore used as more adequate indicators of growth rate. The individuals showing the five lowest and the five highest residuals among the survivors were selected for RNA extraction and gene expression analysis (Figure 1). These groups will be termed S and F groups throughout the manuscript. Average growth rates were 17.1 and 4.1% for F and S clams, respectively.

### Differential expression in the digestive gland

The principal components and cluster analyses did not show any particular clustering of samples (Figures S1, S2). The lists of genes that showed significant (*P* < 0.01) differential expression together with the results of the rank-product test, the fold change and the identification of the gene product through BLAST tools when available (*E*-Value ≤ 1.0e-03) are shown in Table S1. The number of DEGs observed at *P* < 0.01 in the digestive gland was 384, of which 193 were upregulated in F and 191 in S (Table 1). The estimated proportions of false positives (PFP) in these gene sets were 36 and 37%, respectively. At PFP < 0.15, the number of DEGs decreased to 68 probes upregulated in F and 106 in S, respectively (Table 1). Fold change values in this set of genes varied between 1.1 and 77 for genes upregulated in F clams, and between 1.0 and 250 for genes upregulated in S clams (Table S1).

**Table 1:** Summary of the results of the rank-product analysis for gene expression differences between F and S clams, at single-probe *P* < 0.01 and overall 15% probability of false positive (PFP) thresholds.

**Tissue**		**Number of differentially expressed genes**	
		***P* < 0.01**	**PFP < 0.15**
**GILL**			
(13,926 probes)	Total	508	230
	Upregulated in F	301	166
	Upregulated in S	207	64
**DIGESTIVE GLAND**			
(13,994 probes)	Total	384	174
	Upregulated in F	193	68
	Upregulated in S	191	106

**Figure 2:**
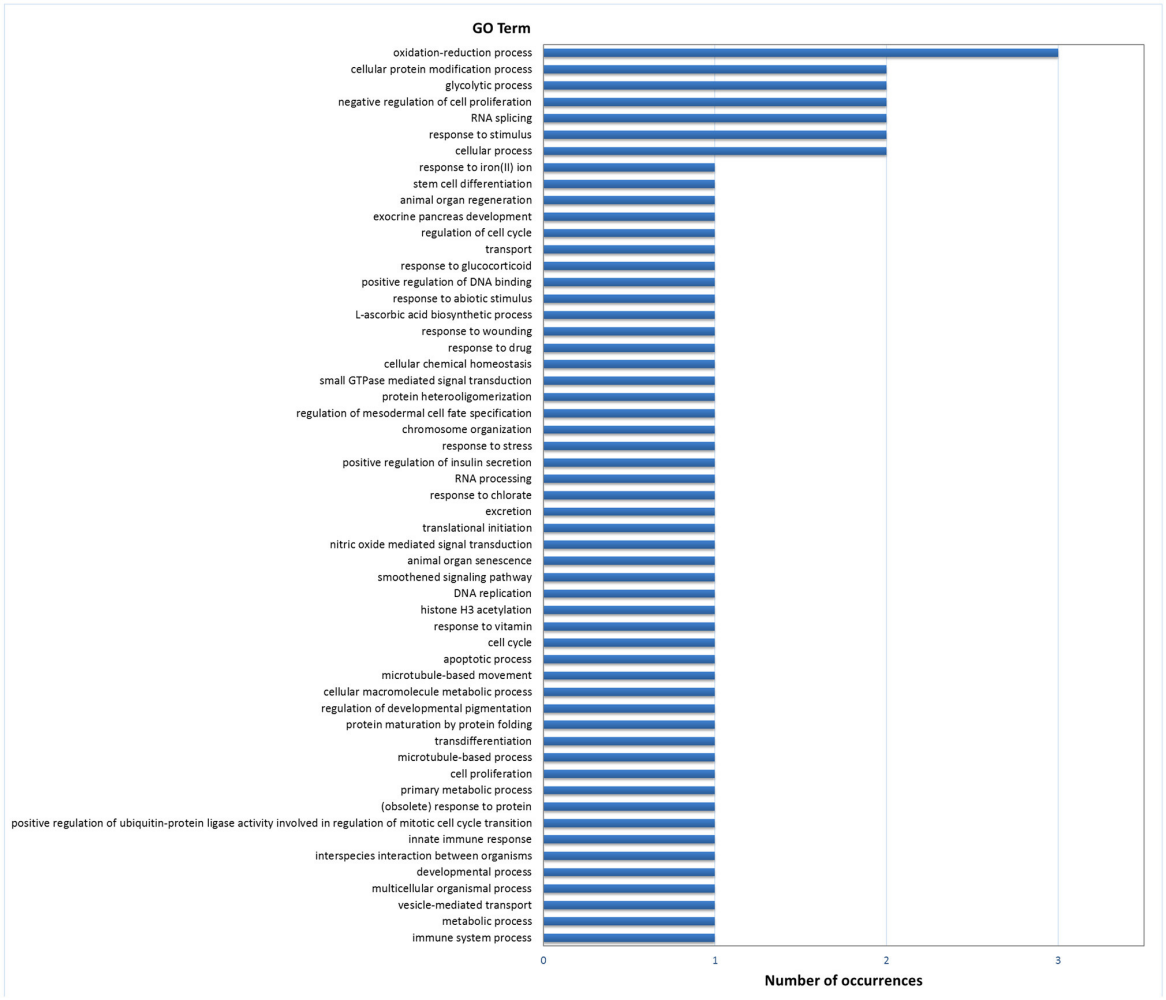
Frequencies of the Molecular Function GO terms in the set of genes that showed significant (PFP < 0.15) differential expression between F and S clams in the digestive gland. The GO terms were treated with REVIGO for grouping terms with semantic similarity.

Gene ontology (GO) analysis of DEGs in digestive gland with Blast2GO resulted in 51 annotated genes in the set of 174 genes with PFP < 0.15, which provided 193 GO terms. The number of terms corresponding to the BP, CC and MF categories after redundancy reduction with REVIGO were 55, 22, and 42, respectively. The most revealing category was BP. This category showed an abundance of terms related with development, cell differentiation and proliferation, carbohydrate metabolism and redox processes (Figure 2). Other less abundant categories include terms related to other growth/related processes such as cell cycle regulation, and categories related with transcription and splicing, signaling, transport and immunity, and defense response.

### Differential expression in the gills

Two samples of gills were excluded due to poor hybridization quality. Gene expression data for a minimum of 2 individuals was available for 13,937 genes in the remaining 8 gill samples (Table S1). The principal components and cluster analyses did not show any particular clustering of samples (Figures S1, S2).

At *P* < 0.01, 508 DEGs were found, of which 301 were upregulated in F and 207 were upregulated in S (Table 1). The estimated proportions of false positives (PFP) in these gene sets were 23 and 33%, respectively. At PFP = 0.15, the number of DEGs in gills was 230, of which 166 were significantly upregulated in F clams and 64 in S clams. Fold change values varied between 10 and 625 for genes upregulated in F, and between 5 and 1,224 for genes upregulated in S.

**Figure 3:**
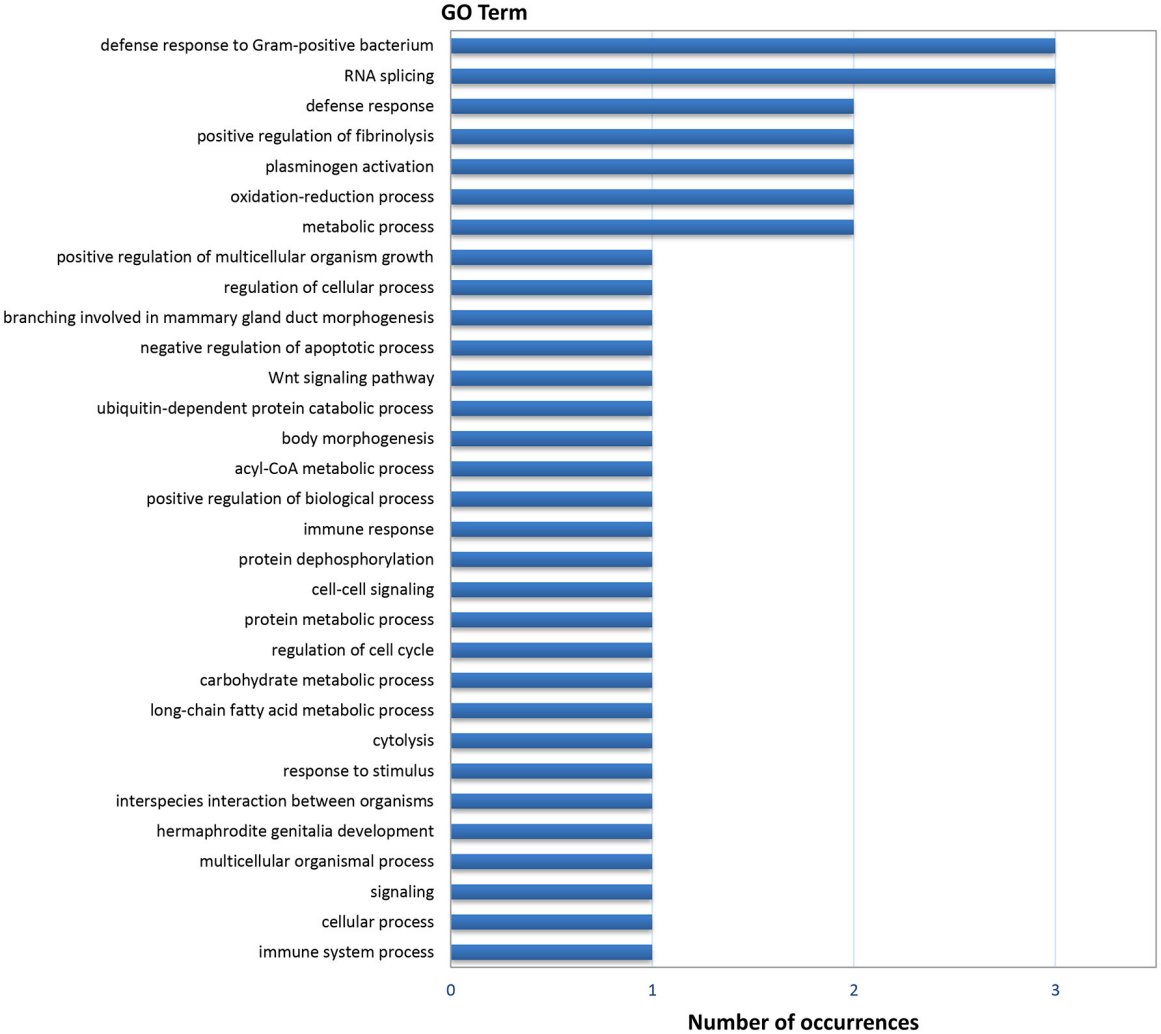
Frequencies of the Molecular Function GO terms in the set of genes that showed significant (PFP < 0.15) differential expression between F and S clams in the gills. The GO terms were treated with REVIGO for grouping terms with semantic similarity.

Gene ontology (GO) analysis of the DEGs resulted in 44 annotated genes, which produced 111 GO terms. The number of terms corresponding to the Biological Process (BP), Cellular Component (CC), and Molecular Function (MF) categories after redundancy reduction with REVIGO were 31, 14, and 28 respectively. As in the case of the digestive gland, the most informative category was BP (Figure 3). GO terms related to immunity and defense against pathogens were very abundant in this category, as well as terms related to protein catabolism and proteolysis. Several terms were related to growth, morphogenesis, development and cell cycle control.

### Differentially expressed genes common to both organs

**Figure 4:**
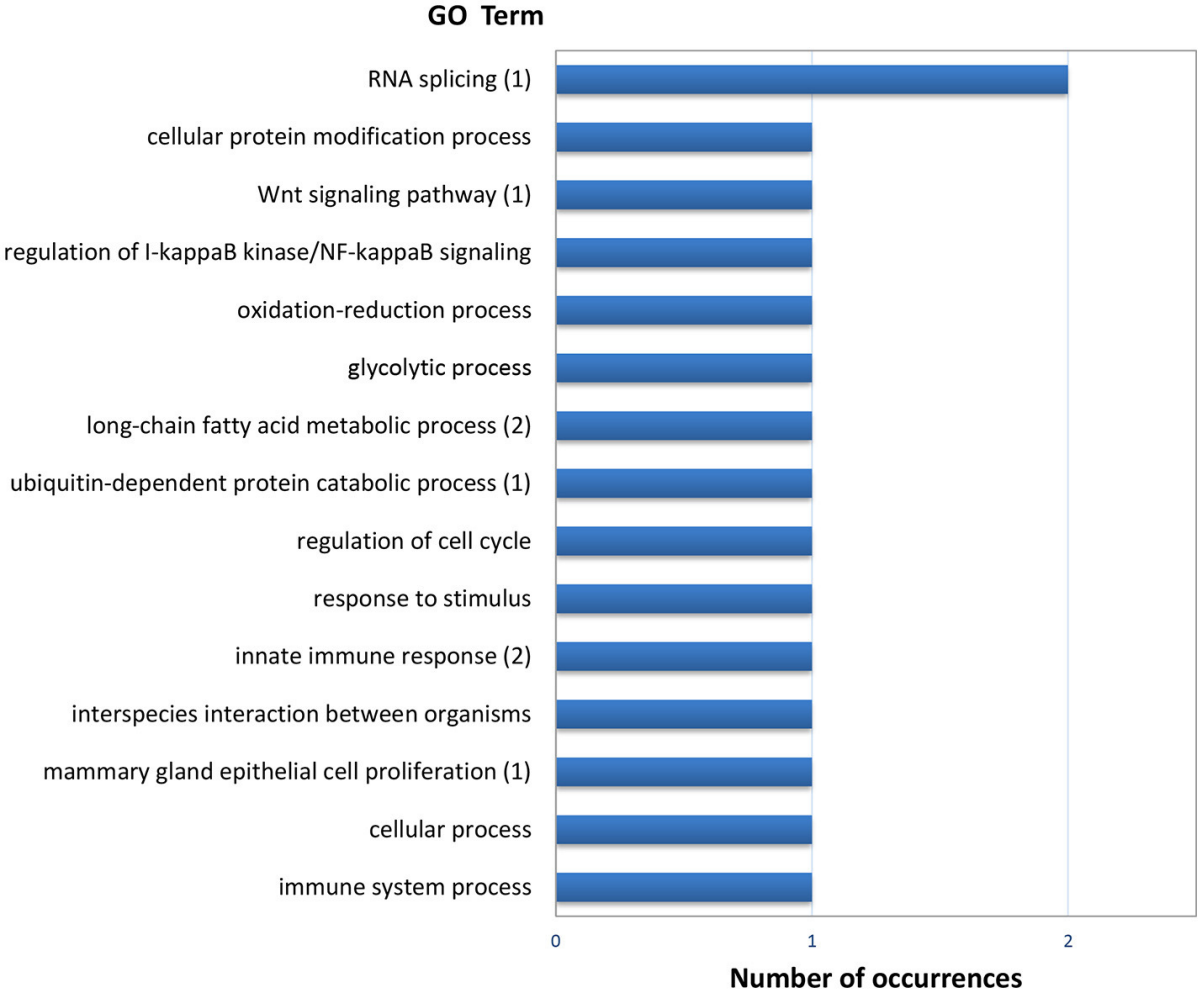
Frequencies of the Molecular Function GO terms in the set of genes that showed significant (PFP < 0.15) differential expression between F and S clams in both the gills and the digestive gland. The GO terms were treated with REVIGO for grouping terms with semantic similarity. The number of terms grouped are given in parenthesis when appropriate.

The list of genes showing significant (PFP < 0.15) differential expression between F and S clams in both gills and digestive gland (Table 2) contains 48 entries, of which 17 were upregulated in F in both organs, 24 were upregulated in S in both organs and 7 showed opposite-sign expression differences in the two organs. Twenty-eight (58%) DEGs produced a significant hit (Eval < 1.0E-03) in Blastx searches. GO terms could be retrieved for 18 genes. The MF terms (Figure 4) reflect the variety of functions represented in this gene set, which can be summarized as cell proliferation, glycolysis, defense response, fatty acid metabolism, protein catabolism, and RNA splicing.

**Table 2:** Genes that showed differential expression in both gills and digestive gland, their protein identity (when available) and groups of clams (F or S) in which they were upregulated.

**Probe #**	**Protein (Blastx)**	**Upregulated (F or S)**	
		**Digestive**	**Gill**
		**gland**	
2673	–	S	S
2981	Tumor necrosis factor member 11	S	S
4141	–	S	S
4567	–	F	S
5630	–	F	F
5663	–	S	F
5744	Zinc finger protein 451	S	S
5955	PREDICTED: similar to ENSANGP00000025755 [Nasonia vitripennis]	F	S
5990	–	F	F
6080	–	F	F
6738	–	S	S
7249	–	S	S
7527	–	S	S
7538	Beta-transducin repeat containing isoform a	S	S
8301	Dentin matrix protein 1	S	S
8807	Protein unq6350 pro21055 homolog precursor	F	F
8897	Hypothetical protein BRAFLDRAFT_202693 [*Branchiostoma floridae*]	F	F
9164	Defensin [*Ruditapes philippinarum*]	S	S
9198	Sialic acid-binding lectin	F	F
9619	Tryparedoxin, putative [*Perkinsus marinus* ATCC 50983]	S	S
9626	Tumor necrosis factor member 11	S	S
9799	Hypothetical protein [*Mercenaria mercenaria*]	F	S
9832	–	F	F
10125	af132070_1ubiquitin-activating enzyme e1	S	F
10215	Hypothetical protein BRAFLDRAFT_70294 [*Branchiostoma floridae*]	S	S
10465	–	S	S
10498	Hydramacin-1	F	F
10655	–	F	F
10979	Plexin-B [*Camponotus floridanus*]	S	S
11140	–	S	S
11326	Glyceraldehyde-3-phosphate dehydrogenase	S	S
11577	Protein unq6350 pro21055 homolog precursor	F	F
11995	Predicted protein [*Populus trichocarpa*]	F	F
12224	–	S	S
12459	–	S	S
12837	–	F	S
12879	Hypothetical protein AaeL_AAEL002481 [*Aedes aegypti*]	S	F
12898	Hypothetical protein BRAFLDRAFT_98726 [*Branchiostoma floridae*]	S	S
12955	Activated rna polymerase ii transcriptional coactivator p15-like	F	F
12976	PREDICTED: cornifelin-like [*Saccoglossus kowalevskii*]	F	F
12990	–	S	S
13062	u2 snrnp auxiliary factor small subunit variant 1	S	S
13072	Hypothetical protein BRAFLDRAFT_72249 [*Branchiostoma floridae*]	F	F
13282	–	F	F
13454	Predicted protein [*Populus trichocarpa*]	S	S
13471	Virion core protein (lumpy skin disease virus)-like protein	F	F
13730	–	S	S
13871	Oocyte-type linker histone b4	F	F

### Differential expression at the growth control gene core

After characterization with BLAST and Blast2GO, a total of 97 probes of our microarray had similarities with proteins of the GCGC (Table S2). Only two were differentially expressed between F and S clams in the two assayed organs (Table 3). One of the DEGs was a putative tumor necrosis factor (probe #2981), a potential effector of the JNK signaling pathway. This gene was upregulated in both the gills and the digestive glands of S clams. The other was a serine–threonin protein kinase with similarity to the ribosomal-protein S6 kinase (#7879), which is activated by the TOR signaling pathway. This gene was downregulated in S clams in the digestive gland. A Fisher exact test indicates that there is no significant enrichment of the GCGC in the set of DEGs observed in the two organs (*P* > 0.05).

**Table 3:** Frequency of differentially expressed genes belonging to the growth control gene core.

**Group**	**No. of genes in microarray**	**Significant differential expression (PFP < 0.15)**
Cell cycle regulators	7	0
Hippo	15	0
Insulin	4	0
Jnk	37	1
Pi3k/Akt	2	0
Ras	19	0
Tor	13	1
TOTAL	97	2

### Other growth-related DEGs

In the digestive gland, 7 DEGs were associated with GO terms related to growth or other functions essential to growth (Table 4). Only one (*Xlox*) was upregulated in F clams. The remaining six genes were upregulated in S clams. These genes coded for proteins with similarity to cell division cycle and apoptosis regulator protein 1 (CCAR), cysteine-rich intestinal protein, sperm nuclear basic protein pl-i isoform plib, F-box/WD repeat-containing protein 1A-like (BTRC), Inhibitor of growth protein 5 (ING5), and DNA replication licensing factor.

**Table 4:** Genes that showed significant (PFP < 0.15) differential expression between F and S clams, and showed GO annotations related to growth or growth-related processes.

	**Probe #**	**Protein (BLASTX)**	**PFP**	**FC**	**Relevant GO terms**
**GILLS**					
**Upregulated in F**	3148	NADH-quinone oxidoreductase subunit B-like	0.106	0.010	P:hermaphrodite genitalia development; P:body morphogenesis; P:nematode larval development; P:positive regulation of multicellular organism growth; P:embryonic development ending in birth or egg hatching
	7955	Protein NOV-like	0.072	0.016	P:regulation of cell growth; F:insulinlike growth factor binding; P: type B pancreatic cell proliferation; P: negative regulation of insulin secretion; P: smooth muscle cell proliferation
**Upregulated in S**	7538	F-box/WD repeat-containing protein 1A	0.004	3.1	P:G2/M transition of mitotic cell cycle P:Wnt receptor signaling pathway P:regulation of IkappaB kinase/NFkappaB cascade P:regulation of cell cycle P:mammary gland epithelial cell proliferation P:positive regulation of ubiquitinprotein ligase activity during mitotic cell cycle
	9771	Transgelin-3	0.131	2.1	P:muscle organ development; P: epithelial cell differentiation
	11101	Baculoviral iap repeat-containing protein 4	0.015	259	P:multicellular organismal development
**DIGESTIVE GLAND**					
**Upregulated in F**	5770	Xlox	0.077	0.5	P:central nervous system development P:organ regeneration P:transdifferentiation P:endocrine pancreas development P:negative regulation of cell proliferation F:protein heterodimerization activity P:response to glucocorticoid stimulus P:response to cytokine stimulus P:positive regulation of cell proliferation P:morphogenesis of embryonic epithelium P:stem cell differentiation P:exocrine pancreas development P:positive regulation of insulin secretion
**Upregulated in S**	557	Cell division cycle and apoptosis regulator protein 1	0.051	2.3	P:cell cycle; P: positive regulation of cell proliferation
	2353	Cysteine-rich intestinal protein	0.039	1.3	P:cell proliferation; P: heart development
	7155	Sperm nuclear basic protein pl-i isoform plib	0.003	2.3	P:developmental process P:multicellular organismal process
	7538	F-box/WD repeat-containing protein 1A-like (BTRC)	0.006	3.0	P: G2/M transition of mitotic cell cycle P:Wnt receptor signaling pathway P:regulation of IkappaB kinase/NFkappaB cascade P:regulation of cell cycle P:mammary gland epithelial cell proliferation P:positive regulation of ubiquitinprotein ligase activity during mitotic cell cycle
	9555	Inhibitor of growth protein 5 (ING5)	0.044	1.6	P:negative regulation of growth P:DNA replication P:negative regulation of cell proliferation
	9922	DNA replication licensing factor	0.127	2.9	P:G1/S transition of mitotic cell cycle; P:regulation of DNA replication initiation P:DNA unwinding involved in replication P:cell cycle

*PFP indicates the probability of false positive associated to the rank product test for differential expression between F and S classes. The fold change (FC) was calculated as the ratio of the average expression in S and F clams. FC > 1 indicates upregulation in S. GO terms were retrieved with Blast2GO (Tables S1, S2)*.

In the gills, five genes with PFP < 0.15 were associated with GO terms which had growth, cell proliferation, cell cycle regulation, cell division or tissue or organ development and differentiation in their definitions (Table 4). Two of them were upregulated in F clams and 3 were upregulated in S clams. These included proteins with similarity to the Nephroblastoma overexpressed (*Nov*) protein, and other proteins with similarity to NADH dehydrogenase iron-sulfur protein mitochondrial precursor, vwc domain-containing protein 3 and scavenger receptor cysteine-rich protein.

### GO term enrichment tests

Results of enrichment tests for GO terms in the digestive gland are provided in Table S3. No significant GO term was significantly enriched (*P* < 0.05) in the subset of DEGs upregulated in F clams, but 14 terms were enriched in the subset of DEGs upregulated in S clams (Table 5). These terms were related to glycolysis, chromatin (assembly and disassembly), and GTPases involved in the Ras signal transduction pathway. No KEGG pathway was significantly enriched in DG at *P* < 0.05 in the sets of genes upregulated in F or S clams, but when F and S were considered together, dre04010 (MAPK signaling pathway) was significantly enriched (*P* = 0.04). The genes associated with the enriched terms in the digestive gland are shown in Table S3. Since the identification of some of these genes through Ensemble *D. rerio* proteins, on which the tests were based (see section Materials and Methods), were supported by low Eval, we checked the identity through Blastx, and the results are also shown in Table S3. The identification through *D. rerio* Ensemble coincided with that obtained through Blastx in almost all cases.

**Table 5:** Results of enrichment test of GO terms carried out with DAVID in upregulated genes (PFP < 0.15) in the digestive gland of S clams.

**GO term**	**Category**	**Count**	**%**	**Fold enrichment**	***P*-Value**	**FDR (%)**
GO:0006091~generation of precursor metabolites and energy	BP	5	9.8	4.7	0.017	19.1
GO:0046578~regulation of Ras protein signal transduction	BP	3	5.9	12.5	0.021	23.2
GO:0006333~chromatin assembly or disassembly	BP	3	5.9	11.6	0.024	26.0
GO:0051056~regulation of small GTPase mediated signal transduction	BP	3	5.9	10.9	0.027	28.9
GO:0006096~glycolysis	BP	3	5.9	10.3	0.031	31.9
GO:0006007~glucose catabolic process	BP	3	5.9	9.7	0.034	34.9
GO:0046365~monosaccharide catabolic process	BP	3	5.9	9.7	0.034	34.9
GO:0019320~hexose catabolic process	BP	3	5.9	9.7	0.034	34.9
GO:0006006~glucose metabolic process	BP	3	5.9	8.7	0.042	40.8
GO:0044275~cellular carbohydrate catabolic process	BP	3	5.9	8.3	0.046	43.7
GO:0046164~alcohol catabolic process	BP	3	5.9	8.3	0.046	43.7
GO:0000785~chromatin	CC	3	5.9	14.5	0.015	12.8
GO:0044427~chromosomal part	CC	3	5.9	7.9	0.047	36.0
GO:0005083~small GTPase regulator activity	MF	3	5.9	9.6	0.035	32.3

*P-values refer to Fisher exact tests. Category abbreviations are as follows: BP, biological process; CC, cellular component; MF, molecular function*.

In the gills, 18 significantly enriched (*P* < 0.05) GO terms were detected in the subset of genes upregulated in F clams and none in those upregulated in S clams After redundancy reduction using REVIGO, 14 terms were left (Table 6). They were related to proteolysis (especially serine-protease activity), defense against pathogens, growth regulation mediated by interactions with insulin-like growth factors, cell adhesion, and biological activity in the extracellular region. No KEGG pathway was significantly enriched in gills (*P* > 0.05). The genes associated with the enriched terms in F clams in gills are shown in Table S4.

**Table 6:** Results of enrichment test of GO terms carried out with DAVID in upregulated genes (PFP < 0.15) in the gills of F clams.

**GO term**	**Category**	**Count**	**%**	**Fold enrichment**	**P**	**FDR(%)**
GO:0040008~regulation of growth	BP	6	5.8	14.8	2.32E-05	0.1
GO:0006508~proteolysis	BP	13	12.6	3.0	6.60E-04	4.3
GO:0006952~defense response	BP	4	3.9	14.2	0.002	13.5
GO:0009617~response to bacterium	BP	3	4.5	16.9	0.012	13.5
GO:0007155~cell adhesion	BP	4	6.1	7.2	0.015	17.0
GO:0022610~biological adhesion	BP	4	6.1	7.2	0.015	17.0
GO:0005576~extracellular region	CC	9	8.7	7.5	5.24E-06	0.02
GO:0005615~extracellular space	CC	3	4.5	26.5	0.004	3.6
GO:0044421~extracellular region part	CC	4	3.9	8.9	0.008	19.8
GO:0005520~insulin-like growth factor binding	MF	5	4.9	30.1	6.86E-06	1
GO:0019838~growth factor binding	MF	5	4.9	25.8	1.57E-05	0.2
GO:0070011~peptidase activity, acting on L-amino acid peptides	MF	12	11.7	4.5	2.81E-05	0.9
GO:0017171~serine hydrolase activity	MF	6	5.8	7.5	8.61E-04	1.3
GO:0008233~peptidase activity	MF	12	11.7	4.2	5.03E-05	1.3

*P-values refer to Fisher exact tests. Category abbreviations are as follows: BP, biological process; CC, cellular component; MF, molecular function*.

## Discussion

Our microarray study of gene expression in F and S clams rendered a set of 356 DEGs, of which 174 appeared in the digestive gland and 230 in the gills. Using this gene set we have tested the main two questions which were at the root of this work. One was the extent of growth-related differential gene expression among organs. The other was the contribution of the set of genes that have been shown to control tissue and organ growth and size in model organisms (the GCGC).

### Growth-associated differential expression at the growth control gene core

Previous studies of growth rate transcriptomics in mollusks have used BLAST searches, gene ontology (GO) and KEGG pathway annotations and enrichment tests to identify individual genes and sets of functionally related genes which underly differences in growth rate among individuals. Some of the genes and enriched GO categories found in these studies were clearly related to what is known about growth physiology. For example, a growth factor receptor has been found to be differentially expressed in abalone (van der Merwe et al., 2011), and enrichment for genes of the KEGG Insulin signaling pathway was found in the pearl oyster (Shi and He, 2014). However, the overwhelming majority of the significant genes, GO terms, and KEGG pathways reported in those studies did not have any obvious relationship with known mechanisms of growth control in animals. In some cases, the DEGs were related to functional aspects that could affect growth. For example, Meyer and Manahan (2010) reported growth-related differential expression of a small cardioactive peptide precursor (SCPb), a neuropeptide involved in the control of muscle contraction that, according to other studies in mollusks, could affect the feeding activity of the animals, and ultimately growth. In other cases, genes related to the production of energy, especially proteins of the mitochondrial respiratory chain, have appeared as differentially expressed in growth classes (Meyer and Manahan, 2010; Valenzuela-Miranda et al., 2015). However, these were notable exceptions. One potential partial explanation for this paradox is that the annotation of mollusk genes is difficult due to their evolutionary distance to vertebrates, which are the main source of genetic functional information for GO analyses in animals. In fact, most transcriptomic studies of growth in mollusks report a low rate of transcript annotation, and the percent of identified proteins among DEGs is usually below 40% (Meyer and Manahan, 2010; Choi et al., 2015; Valenzuela-Miranda et al., 2015). The low rate of identified and annotated genes can have the consequence of lowering the power of enrichment tests for GO categories and KEGG pathways, resulting in the misidentification of functionally relevant sets of genes.

A possible way to overcome this limitation is the one we have used in this work. Genes relevant for growth control have been identified from the relevant literature (the GCGC) and their contribution to the set of growth related DEGs in our clams has been specifically tested. In our study, only two out of the 97 growth control core genes that were present in the microarray showed differential expression between F and S clams. Valenzuela-Miranda et al. (2015) reported differential expression in one gene of the GCGC out of the 32 genes showing the largest differences in growth-associated expression in the red abalone. All these results indicate that this set of genes does not contribute importantly to the growth-associated gene expression variability observed in mollusks. However, we should be cautious about this result. Some specific features of the experiment could be limiting our power to detect significant differential expression. One is that our analysis is based on a small number of individual samples. This could lead to low detection power if absolute expression values of the growth control core genes vary too much among individuals. For example, wide variation in the expression of genes related to insulin signaling has been observed in humans (Wang et al., 2015). A deep characterization of growth-associated gene expression patterns using more powerful techniques, such as RNA-seq, will give a definitive answer to this question. Another complication is that our clam population suffered important mortalities at the end of the experiment. The causal agent of this mortality, which is unknown, could have affected the expression of some growth control genes.

In spite of the negative result of the enrichment test for the GCGC as a whole, some of our results support the importance of some members of that gene set in accounting for growth rate differences. Four out of the 12 genes that showed differential expression and had associated GO terms related to growth were functionally related to the IIS pathway or to other core transduction cascades (Table 4 and subsections below). The results of GO term enrichment also showed the importance of these routes in our results, as the GO terms “regulation of growth,” “insulin-like growth factor binding,” and “growth factor binding” were enriched in the gills and “regulation of Ras protein signal transduction” was enriched in the digestive gland. But as a rule the majority of the observed DEGs in this study are part of other routes which are also interesting from the point of view of characterizing the physiology of growth differences in clams. All them will be discussed below, separately for each organ.

### Organ–specific and shared DEGs

Only 12% of the DEGs were common to digestive gland and gills. One obvious source of the observed gene expression differences between organs could be their different functional roles. While big differences in DEGs between organs could be anticipated, there are other potential sources of gene expression differences that should also be considered. One is the experimental error associated to the experiment performance. Due to organizative reasons, the microarray hybridization of the two organs was separated 6 months in time, so they should be considered as different experiments. Although the contribution of this source of variance cannot be estimated, we can safely assume that it is small, as it has been shown that the correlation across experiments based on the same microarray is usually higher than 0.9 (Chen et al., 2007). Another source of divergence is the difference in the level of expression of a gene in the two organs. One gene could exhibit a much higher expression difference between F and S clams in one organ than in the other, resulting (everything else being equal) in higher power for the statistical test to detect it in the organ that exhibits the highest difference. If this situation were common, we should expect that the *P*-values for the statistical test of differential expression would be correlated in the two organs. Pearson correlation coefficients of P (F > S) in gills and digestive gland for the list of significant genes of gills (Table S1) was 0.54 (*P* < 0.001). A similar result was obtained for the list of significant genes of the digestive gland. This result means that this source of differences is real, although the correlation is not high and therefore it should have low impact on the results. In conclusion it seems reasonable to accept that the great majority of the observed differences in DEGs between organs are due to their different functional roles.

The functional characterization of DEGs common to both organs through gene ontologies was achieved in 18 of the 48 cases only, but even this limited information is very revealing, and points to specific functions: transcription regulation, RNA splicing, sugar metabolism, protein catabolism, biosynthesis of long chain fatty acids, and immunity and host defense (Figure 4). Differential expression common to both organs affects therefore very central aspects of the organism and cell activity and is not related specifically with growth control genes. Since there is a certain limitation of power, as indicated by the correlation analysis of the P (F > S) values, these functions are presumably only part of the set of common functions affected by growth-related differential expression in clams.

However, the majority of DEGs showed organ specificity. BLAST analysis and enrichment tests for GO terms revealed several molecular features in which the two organs differ. The observed differences unveil organ-specific molecular aspects of growth-rate variation among individuals, many of which are shown for mollusks for the first time.

### Growth-associated differential expression in the digestive gland

In mollusks, insulin-like peptides have been characterized as the main growth regulatory molecules (Taylor et al., 1996). In *Drosophila*, different organs can secrete different types of insulin (Andersen et al., 2013). In mollusks, insulin-like peptides are secreted by cerebral and visceral ganglia (Hamano et al., 2005), but also specific cells involved in secretion of insulin-like substances have been detected by histological techniques in the intestine of bivalves (Fritsch et al., 1976; Plisetskaya et al., 1978). Several results of our study point to growth-related gene expression differences in genes functionally connected to the IIS axis, with outstanding examples in the digestive gland. The first one is *Xlox*, a gene that is fundamentally related to insulin production in vertebrates. *Xlox* is known as *PDX1* in vertebrates and insulin promoter factor 1 (*IPF1*) in humans. *Xlox* pertains to the *ParaHox* gene family that controls the digestive tract organogenesis in animals. The gene is involved in midgut patterning in mollusks, and its expression does not cease at the end of the larval period, as it does in the case of other *ParaHox* genes (Samadi and Steiner, 2010). In vertebrates, the expression of *Pdx1* is necessary for the development of β-cells in pancreas, which are responsible for insulin production (D'Amour et al., 2006). As expected if *Xlox* expression induced higher insulin levels, and therefore increased insulin-promoted growth, *Xlox* showed increased expression in the digestive gland of F clams (Table 4). Another DEG connected to the IIS axis is the cell division cycle and apoptosis regulator protein 1 (CCAR1) gene, which was upregulated in the digestive gland of S clams (Table 4). CCAR1 interacts with many other proteins, such c-myc, p-53, and cell cycle regulators, resulting in a variety of roles affecting many cell processes and functions, but usually by maintaining the balance between apoptosis, proliferation, and differentiation. D'Amour et al. (2006) have demonstrated that CCAR1 is necessary for the differentiation of endocrine pancreatic cells in vertebrates, which are responsible for the production of insulin and other hormones, and Lu et al. (2012) showed an antagonistic effect of CCAR1 and *Pdx* (the mammal version of *Xlox*) in a pancreatic cell line. The observation of differential expression of *Xlox* and CCAR in opposite directions in the digestive gland of clams in this study suggests that the role of these two genes in bivalves could be similar to that described in vertebrates.

One of the transduction cascades that are included in the GCGC is the Ras/Raf/MAPK. While no DEG was observed for genes in this cascade in our study, GO enrichment tests rendered significant enrichment for gene ontologies associated to GTPases involved in Ras signal transduction in the digestive gland of S clams (Table 5). GTPases are essential enzymes of the Ras/Raf/MAPK. This significant result was due to the upregulation of three genes, which coded for titin (connectin), epithelial cell transforming factor (ECTF), and G protein-coupled receptor kinase interacting protein ArfGAP 2b (Table S3). Titin is a protein of striated muscle and condensed chromosomes (Machado and Andrew, 2000). ECTF is involved in the control of cytokinesis (Tatsumoto et al., 1999), and ArfGAP is involved in the regulation of membrane traffic and cytoskeleton remodeling (Randazzo and Hirsch, 2004). It is difficult to figure out where in the complex network of interactions of Ras/Raf/MAPK with other pathways can these proteins be located (Keshet and Seger, 2010). Nevertheless, this result shows that important aspects of growth regulation mediated by the Ras/Raf/MAPK cascade can appear at a diverse array of cellular settings.

The remaining DEGs found in the digestive gland were not immediately related to the genes of the growth control core. However, many of them are probably influenced by molecular mechanisms downstream of the main transduction cascades of the GCGC, mediated by transcription factors that stimulate or repress the synthesis of growth factors or other proteins (Gokhale and Shingleton, 2015). The set of DEGs found in the digestive gland was enriched in genes related to “chromosomal part,” “chromatin,” and “chromatin assembly and disassembly,” which refer to the transcriptional activity (Table 5). Moreover, our search for growth-related GO terms in the set of DEGs in this organ found one gene which was upregulated in the digestive gland of S clams and coded for an Inhibitor of growth protein 5 (ING5) (Table 4). ING5 is involved in transcription regulation and chromatin remodeling (Shiseki et al., 2003). In addition, 13 other DEGs with associated GO terms related to transcription were found in the digestive gland (Table S1). These include RNA polymerase subunits and coregulators. All together, these results indicate the importance of the changes in transcriptional activity related to individual growth differences in the digestive gland in clams.

One of the final effects of the GCGC is the regulation of cell growth, DNA replication, cell cycle control, cell division, and cell proliferation (Gokhale and Shingleton, 2015). Several genes related to some of these processes showed differential expression in the digestive gland in this study. Two genes involved in DNA replication were upregulated in S clams (Table 4). One was the DNA replication licensing factor *mcm5*, which codes for a subunit of the MCM2-7 complex, a putative replicative helicase essential for ensuring a single round of DNA replication per cell cycle in eukaryotic cells (Tye, 1999). The other was the already mentioned ING5protein, which interacts with the MCM complex and plays an essential role in DNA replication (Doyon et al., 2006). One gene related to cell cycle control with similarity to the F-box/WD repeat-containing protein 1 (FBXW1), also known as beta-transducin repeat containing protein (BTRC), was upregulated in gills and digestive gland in the slow growing clams (Table 4). It has been shown that FBXW1 participates in the degradation of CDC25A protein phosphatase, which turns off CDK1 and therefore keeps the cell in the G1 phase (Jin et al., 2003). This role could provide a link between higher FBXW1 expression and slow growth rate in clams by limiting the cell proliferation rate.

Growth is generally associated to an increase of protein synthesis (Fraser and Rogers, 2007). In other studies in mollusks growth rate was also positively associated with the expression of proteins implicated in protein synthesis, including many ribosomal proteins (Meyer and Manahan, 2010; van der Merwe et al., 2011; Choi et al., 2015). In our microarray, 380 probes were identified as genes related to this important function, but only 3 in the digestive gland showed differential expression between F and S clams. These genes coded for 60s ribosomal protein L34, ubiquitin-60s ribosomal protein l40, and a ribosome biogenesis protein rpf2 homolog (Table S1). While these results indicate that changes in the expression level of some important enzymes involved in the synthesis of ribosomal proteins are associated with growth rate, they are also contrasting with some of the published studies, which showed a much higher number of DEGs coding for ribosomal proteins. In particular, Meyer and Manahan (2010) reported that roughly half of the genes that showed differential expression between growth classes in oysters were ribosomal proteins. The study of Meyer and Manahan (2010) was designed to detect the influence of heterosis in growth and the material analyzed was oyster larvae resulting from a hybrid cross between inbred lines and larvae from pure crosses of the parental lines, while wild clams taken from a random-mating population were analyzed in our study. These differences between the two studies suggest that the faster growth rate caused by heterosis in crossbred oyster larvae could be more dependent on protein biosynthesis systems that the differences in growth rates observed among wild adult clams.

Energy supply (ATP, glucose) has an important incidence on growth rate (Gokhale and Shingleton, 2015). Therefore, growth-related variability in the expression levels of the genes involved in the biochemical pathways responsible for sensing these factors, or for regulating growth according to the levels of these factors, is expected, especially in organs directly related with energy storage and mobilization of energy reserve compounds such as the digestive gland. A significant enrichment of GO terms related to glucose metabolism and electron transport chain, such as “Generation of precursor metabolites and energy” and other related to glucose metabolism, was observed in this organ in the present study (Table 5). Upregulation of a gene coding for a subunit of the NADH dehydrogenase, a member of the enzymatic complex responsible for electron transfer in the inner mitochondrial membrane and ATP production, was included in this list. Meyer and Manahan (2010) also found differential expression at the NADH dehydrogenase in fast-growing hybrid oyster larvae. Growth-related differential expression has been reported also for other mitochondrial respiratory chain components in mollusks, such as the ATP synthase (Meyer and Manahan, 2010) and the cytochrome c oxidase (Valenzuela-Miranda et al., 2015). Moreover, three genes that coded for two enzymes involved in glycolysis, namely glyceraldehyde-3-phosphate dehydrogenase (G3PDH) and phosphoglycerate mutase and one enzyme acting in the Krebs cycle and the malate shuttle (malate dehydrogenase), were also upregulated in S clams. NADH dehydrogenase and G3PDH were also overexpressed in the gills of S clams, suggesting that upregulation of these genes could be a feature of S clams in the two organs. These results point to high energy requirements in slow growing clams, which are enhancing the expression of relevant genes related to energy production to meet these requirements. Alternatively, the observed differential expression patterns could be reflecting an inbalance in the expression of the genes related to energy metabolism which would result in physiologic impairment and slow growth.

### Growth-related gene expression differences in the gills

Evidence for DEGs related to the IIS axis in the gills of clams has come from the enrichment analyses. The results of these tests indicated significant enrichment of the terms “growth” and “insulin growth factor” in the gills. However, a close examination of the genes ascribed to these GO terms indicates that these are not genes participating directly in the IIS axis (Table S4). They represent a set of seven genes which showed upregulation in F clams in the gills and have similarity to five zebrafish proteins: cysteine-rich angiogenic inducer 61 like 1 (cyr61), tenascin C, WNT1 inducible signaling pathway protein 1b (wisp1b), cysteine rich transmembrane BMP regulator 1 (chordin-like)(crim1), and connective tissue growth factor a (Ctgfa). BlastX searches provided more accurate hits for four of these genes, which were identified as nephroblastoma overexpressed protein (NOV)-like (two cases), collagen IV (two cases), and a serine-protease in the case of the gene with similarity to zebrafish tenascin. Tenascin is actually a serine-protease, so this identification was essentially the same. Interestingly, cyr61, Ctgfa, wisp1b, and NOV belong to the same protein family, namely the CCN family of secreted proteins (Perbal, 2013). These are cystein-rich secreted extracellular matrix proteins (Leask and Abraham, 2006). CCN are multimodular proteins, which have an insulin growth factor-binding protein (IGFBP)-like module that allows for interactions with insulin growth factor (IGF) receptors and IGF binding proteins. They also have a module with similarity to the Von Willebrand Factor Type C repeat (VWC), which is also present in collagen (Planque et al., 2003), and could be the cause that some of the clam genes with similarity to zebrafish CCN protein produced also Blastx hits to collagen. CCN proteins are involved in signaling and regulation of various biological functions. In vertebrates, where these genes have been most studied, these functions include adhesion, extracellular matrix remodeling, cell proliferation, skeletal development, chondrogenesis, angiogenesis, and wound repair (Holbourn et al., 2008; Chen and Lau, 2009). The association of different levels of expression of these proteins with growth rate differences in clams suggest an important influence of biological regulation processes taking place in the extra cellular matrix in the gills of these organisms. This view is supported by the significant enrichment for GO terms “extracellular region” and “extracellular space,” which record the differential expression of CCN proteins in addition to other extracellular proteins (Table S4).

The most outstanding case of specific DEGs in gills is the high number of genes coding for peptidases or other proteinases (18), as compared to only 2 in the digestive gland. This resulted in significant enrichment of GO terms related to proteolysis, peptidase activity and hydrolase activity (Table 6). There were also 8 DEGs coding for peptidase inhibitors in gills. Antimicrobial activity has been ascribed to some peptidases in mollusks (Venier et al., 2011; Allam and Raftos, 2015), and some specific proteinases such as the lysozyme, have usually a defensive role. The microarray contained nine genes that showed similarity to lysozyme, but only one showed significant differential expression in gills (Table S1, probe #13229). Proteolytic cascades have also important roles in several biological processes related with immunity and defense against pathogens (Cerenius et al., 2010). Therefore, while it is possible that part of the proteinase differential expression is related to defense against pathogens, other functions seem also probable. Proteolytic cascades also have an important role in degrading enzymatic proteins to control precisely their activity in time and space in the cell (Rodríguez et al., 2010). This function is achieved by digesting inactive enzyme forms (propeptides or zymogens) and by degrading active forms. Some proteins which were upregulated in the gills could be regulated in this way. As an example, proteins in the CCN family discussed above are known to interact with proteinases while acting in the extracellular matrix (Chen and Lau, 2009). CCN proteins are mosaics of four modules united by a protease-sensitive region, and one or more of their specific modules are often detected alone (Leask and Abraham, 2006). The upregulation of proteinases in gills, therefore, is in agreement with the upregulation of CCN proteins observed.

GO enrichment tests were significant for the terms “defense response” and “defense to bacterium” in the gills, which referred to a group of 4 proteins including toll-like receptor 8b, serum amyloid A and 2 peptidoglycan recognition proteins. This reflects the high abundance of DEGs related to host defense responses against bacteria or parasites in both the gills and the digestive gland. More than 130 DEGs in both organs had “immunity” or “defense” as associated GO terms. These genes included a high number of the most abundant classes of proteins related to defense against pathogens and immunity, such as lectins, c1q containing proteins, defensin, lysozyme, plexin, cornifelin, hemagglutinin amebocyte aggregation factor, and tumor necrosis factor (Venier et al., 2011; Song et al., 2015). Previous studies of growth-related gene expression in mollusks have also reported differential expression for many disease response genes (Shi and He, 2014; Choi et al., 2015). The high abundance of disease-related DEGs in our study suggest that differences in the level of gene expression between F and S clams at certain genes could reflect differences in fitness among individuals, and that fitter clams are able to grow faster and, at the same time, to fight more effectively against disease.

## Conclusions

Transcriptional studies of growth rate variability in mollusks usually have found a large amount of DEGs. The set of genes of interest can be narrowed down by examining different organs separately and constructing specific hypothesis for the trait of interest based on available physiologic and molecular information. We have tested the hypothesis that gene expression variability associated to growth rate variation might be concentrated on the set of genes that has been shown to control tissue and organ growth and body size in model organisms, which we term the GCGC. The specific examination of this hypothesis is especially interesting, because these gene set has a good experimental support as to its involvement in growth control, and especially because its confirmation would facilitate the study of growth in many organisms that are not well characterized at the genetic and genomic levels by restricting the analysis to a relatively well known set of genes. Our results indicate that genes in the growth control core do not contribute importantly to the growth-associated gene expression variability observed in mollusks. In spite of this negative result, the fact that a few growth control genes showed differential expression indicates that they can be involved in growth rate variability to an extent still unknown. Given their demonstrated role in growth control in model organisms, we suggest that they are routinely examined in transcriptomic studies of growth rate.

We also have characterized the set of genes that are differentially expressed in F and S clams. These results reinforce the role of insulin-mediated processes in growth variation. However, the differentially expressed insulin-related genes found are not the main genes associated to the insulin/insulin-like growth factor signaling pathway (IIS), but other genes that interact functionally with the IIS such as the CCN proteins. In other cases, they are genes with a fundamental role in the organogenesis and differentiation of the cells responsible for insulin production in the digestive gland (*Xlox*, CCAR).

Finally our study has also revealed some shared and specific functions of the gills and the digestive gland, related to growth-rate variation. In the digestive gland, genes related to transcriptional activities and Ras signaling are differentially expressed, which is in line with the diverse array of functions that the digestive gland may have (Röszer, 2014; Wang et al., 2014). Moreover, differential expression was also detected in this organ at genes related to energy metabolism, which also fits the important role of the digestive gland in energy storage and mobilization of reserve compounds. At the gills we have described for the first time differential expression at the CCN proteins of the extracellular matrix, which have a relevant role in cell differentiation at some tissues in vertebrates (Perbal, 2013). Unexpectedly, we also observed in the gills differential expression for proteinases and peptidases. These patterns should be confirmed in new studies. Our results suggest that a more detailed characterization of the modulation of growth through differential gene expression patterns in the different organs of mollusks by using more powerful techniques such as RNA-seq or RNA interference will be worth pursuing.

## Data accessibility

Gene expression analyses were performed using a 8X15K Agilent oligo-DNA microarray platform deposited in the GEO database under accession number GPL23511. Microarray raw and normalized fluorescence values were deposited in the GEO database (http://www.ncbi.nlm.nih.gov/geo) under accession number GSE99243 and GSE99244.

## Author contributions

CS, LB, MM, and RL designed the study; CS and RL reared the clams and measured growth; CS, DC, and MM performed the molecular work; LB, MC, and TP contributed reagents; CS and MM analyzed the data; CS and MM wrote the paper; all authors read the paper draft and contributed their comments and discussions.

### Conflict of interest statement

The authors declare that the research was conducted in the absence of any commercial or financial relationships that could be construed as a potential conflict of interest.
