# Supplementary material for: A Microarray Study of Carpet-Shell Clam (Ruditapes decussatus) Shows Common and Organ-Specific Growth-Related Gene Expression Differences in Gills and Digestive Gland
Source: Front Physiol. 2017 Nov 28;8:943. doi: 10.3389/fphys.2017.00943 (PMC5712350; doi:10.3389/fphys.2017.00943)

Supplementary Presentation.- Figures S1 and S2

A microarray study of carpet-shell clam (*Ruditapes decussatus*) shows common and organ-specific growth-related gene expression differences in gills and digestive gland.

Carlos Saavedra, Massimo Milan, Ricardo B. Leite, David Cordero, Tomaso Patarnello, Leonor Cancela and Luca Bargelloni

Figure captions

Figure S1. Results of average linkage clustering based on Pearson correlation coefficients for the gill and the digestive gland expression data.

Figure S2. Results of the principal component analysis of the expression data in gills and digestive gland. Only the plots corresponding to the three first commponents are shown.

Fig. S1

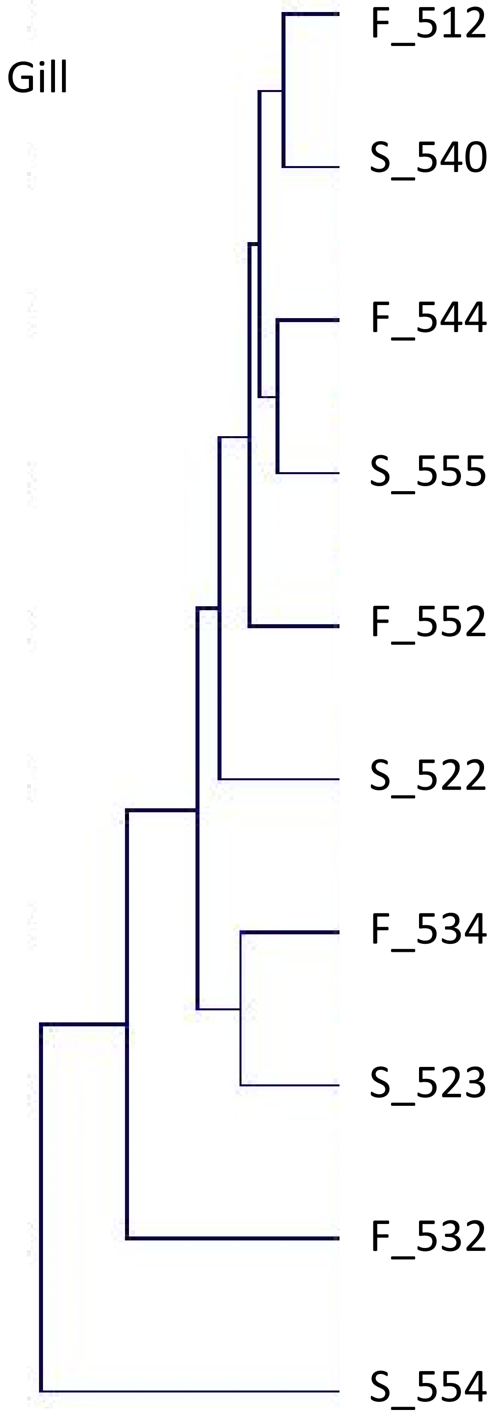

Gill
F_512
S_540
F_544
S_555
F_552
S_522
F_534
S_523
F_532
S_554

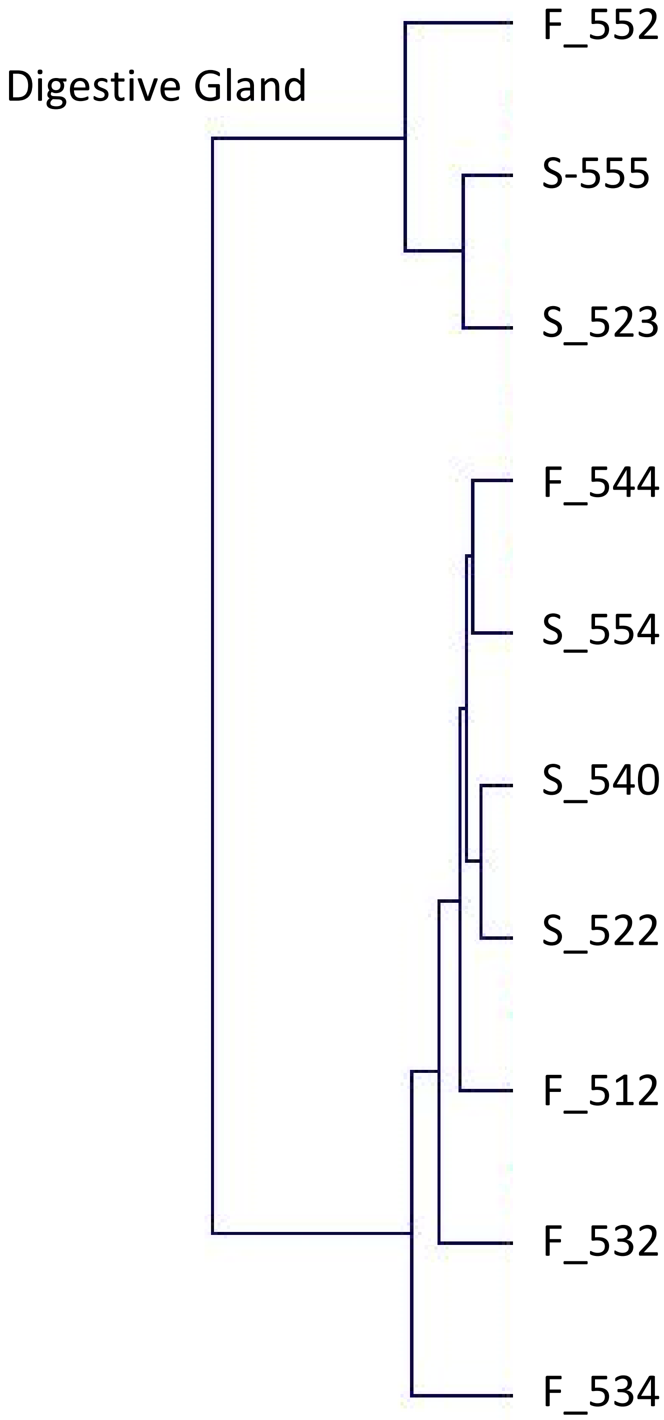

Digestive Gland
F_552
S-555
S_523
F_544
S_554
S_540
S_522
F_512
F_532
F_534

Fig. S2

Gills

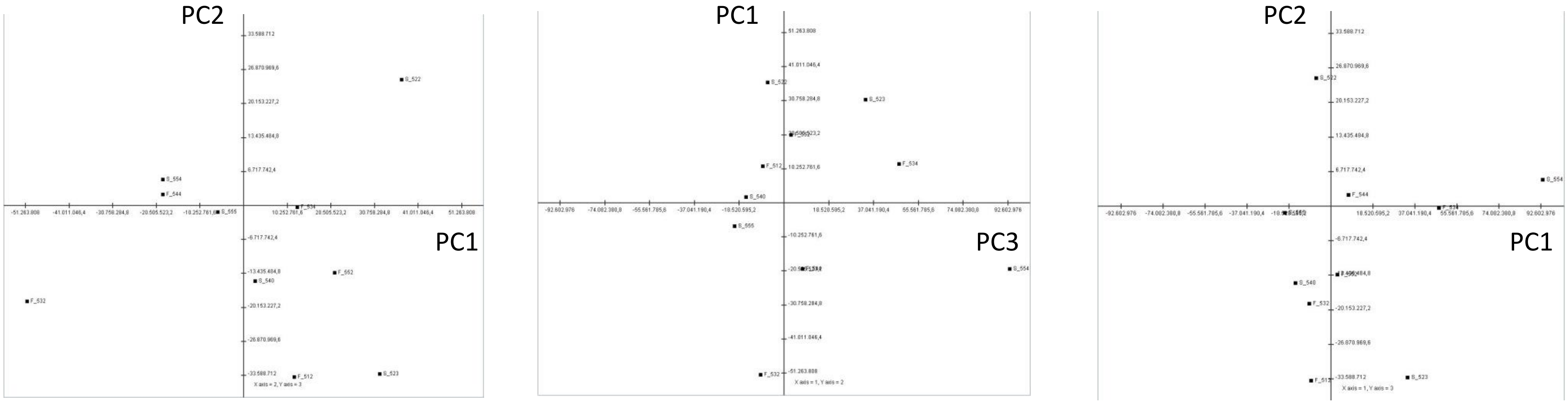

Digestive gland

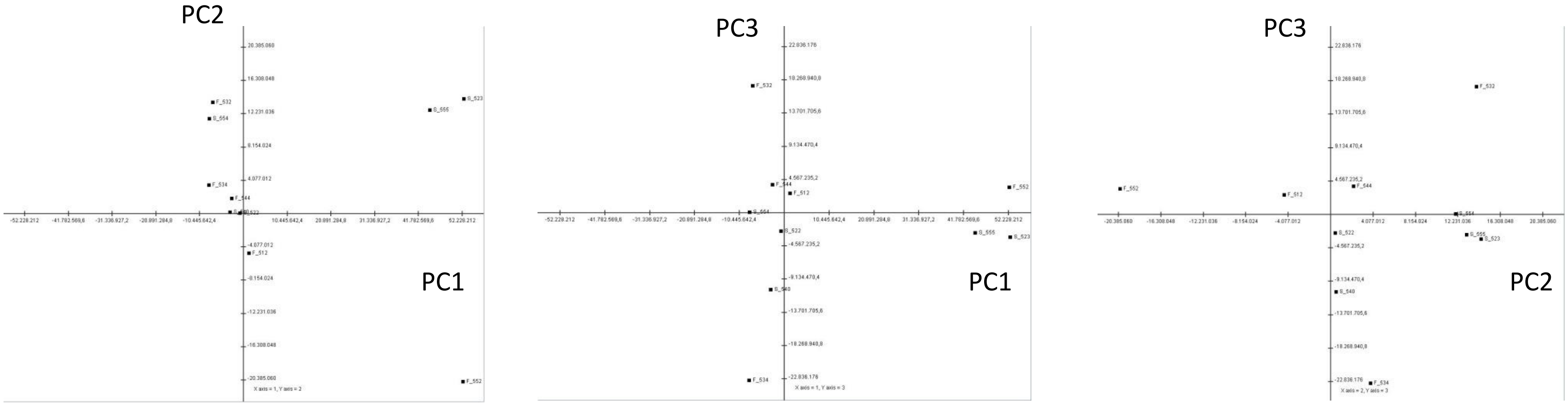

Supplement: Supplementary file 2 [file Presentation1.pdf]
